# Reimagining a summer research program during COVID: Strategies for enhancing research workforce diversity

**DOI:** 10.1017/cts.2022.371

**Published:** 2022-02-28

**Authors:** Brenda L. Eakin, Phillip A. Ianni, Christy Byks-Jazayeri, Vicki L. Ellingrod, Susan J. Woolford

**Affiliations:** 1 Michigan Institute for Clinical and Health Research, University of Michigan, Ann Arbor, MI, USA; 2 College of Pharmacy, University of Michigan, Ann Arbor, MI, USA; 3 Department of Pediatrics, University of Michigan, Ann Arbor, MI, USA

**Keywords:** Diversity, inclusion, health disparities, remote learning, research training

## Abstract

Well-designed, accessible short-term research training programs are needed to recruit and retain underrepresented persons into clinical and translational research training programs and diversify the workforce. The Michigan Institute for Clinical and Health Research developed a summer research program, training over 270 students in 15 years. In response to the 2020 COVID-19 pandemic, we pivoted swiftly from an in-person format to a fully remote format. We describe this process, focusing on factors of diversity, equity, and inclusion including enabling student participation in remote research activities. We collected data about students’ learning experiences since the program’s inception; therefore, we could evaluate the impact of remote vs. in-person formats. We examined data from five cohorts: three in-person (2017–2019; *n* = 57) and two remote (2020–2021; *n* = 45). While there was some concern about the value of participating in a remote format, overall students in both formats viewed the program favorably, with students in the remote cohorts rating some aspects of the program significantly more favorably. In addition, more students who identified as Black or African American participated in the remote format than in the in-person format. We describe lessons learned from this unprecedented challenge and future program directions.

## Introduction

In recent years, there has been an increasing need to diversify the clinical and translational science workforce. In 2019, the National Institutes of Health released updated guidelines encouraging institutions to enhance the diversity of their workforce, stating that “scientists and trainees from diverse backgrounds and life experiences bring different perspectives, creativity, and individual enterprise to address complex scientific problems” [1]. To achieve greater diversity in the clinical and translational research (CTR) workforce, efforts have been made to develop training programs that are intended to recruit individuals from underrepresented populations [2–7]. However, the proportion of underrepresented minority researchers in the CTR workforce continues to be smaller than that of the US population at large [8]. Therefore, it is critically important to establish substantive multidisciplinary research training to recruit and retain underrepresented minority individuals as well as those from disadvantaged backgrounds into CTR programs.

In 2007, the Michigan Institute for Clinical and Health Research (MICHR) at the University of Michigan developed a CTSA-sponsored immersive, 3-month summer research program to provide graduate students from lower-resourced institutions with a structured CTR experience. In 2012, the program began intentionally recruiting from groups traditionally underrepresented in translational research. Studies related to other summer research programs have demonstrated that this type of short, immersive experience has many benefits, including an increase in students’ self-reported interest in research and research skills [9–12], having an increased publication rate [13], and an increased likelihood of students pursuing a research career [7,10,14].

From 2007 to 2019, MICHR’s summer research program was implemented as an in-person, mentored research experience. However, like many other CTSA hubs, in 2020 the COVID-19 pandemic necessitated an unanticipated and swift change in program format. Although previous work has described the impact the COVID-19 pandemic has had on CTR trainees and scholars [15,16], few studies have evaluated the effects of pandemic-related changes to the curriculum of a CTR training program [17]. In this paper, we will describe the process of quickly converting an established, in-person program to a comprehensive remote format, focusing on factors of diversity, equity, and inclusion. We will compare the impact of these changes by examining pre- and post-COVID student programmatic experiences and describe lessons learned from this unique challenge.

## Methods

### The Summer Research Program

The MICHR summer research program was developed in 2007 to provide students in master’s and health professions degree programs with a participatory experience in translational research. In 2012, the program was restructured to concentrate on health disparities research. The program attracts students from across the US and Puerto Rico who have diverse research interests and backgrounds. Students accepted into the program participate in activities focused on interdisciplinary and collaborative work in translational and health disparities research.

This short-term program is offered from June through August each year. As part of their application, students provide a brief description of their research interests, which assists the program manager in matching them with a faculty member at the University of Michigan who shares their research interests. Faculty mentors in all years of the program were given the opportunity to interview prospective students before agreeing to work with them.

Students work approximately 35 hours a week with their assigned faculty mentor on an ongoing research project and participate for approximately five hours a week in a structured curriculum that explores substantive methodological and career topics related to health disparities and translational research. The curriculum is aligned with seven of the CTSA core thematic areas [18] including 1) CTR questions, 2) study design, 3) regulatory support and knowledge, 4) responsible conduct of research, 5) translational teamwork, 6) leadership, and 7) community engagement (see Table 1). The structured learning components (e.g., activity-based seminars, journal club, group projects, and community-based site visits) provide students with hands-on experience in a range of health disparities and translational research activities that allow for consistent opportunities for interdisciplinary learning. The program also includes required training in the protection of human subjects and responsible conduct of research and multiple opportunities to learn and practice scientific communication skills. At the beginning of the program, students complete a short, 5-station research-focused objective structured clinical exam (R-OSCE) to assess their health disparities and translational research knowledge and skills. An in-depth discussion of this instrument is described elsewhere [19]. Students are encouraged to discuss the results of the exam with their mentors and use them when developing their individualized development plans for the program.

**Table 1.** Program curriculum

Program week	Curriculum topic (presented 2017–2021 unless otherwise noted)	Competency covered (ctsacentral.org)
Weeks 1–4	Orientation, mentor training, human subjects’ protections, and HIPAA training	VIII. Clinical research interactions (regulatory support and knowledge)
	Mentored research projects	IV. Research implementation; XI. Translational teamwork; XII. Leadership
	Introduction to health equity and social determinants of health	X. Cultural Diversity XIV. Community engagement
	Making the most of mentoring relationships	XI. Translational teamwork
	Writing abstracts	IX. Scientific communication
	Writing specific aims	I. Clinical and translational research questions
	Conducting and obtaining valid informed consent	VIII. Clinical research interactions (RCR)
	IRB experience (2017–2019)	VIII. Clinical research interactions (RCR)
	Conducting and applying research – team science	III. Study design; XI. Translational teamwork; IX. Scientific communication
	Interpreting biostatistics	VI. Statistical Approaches
	Weekly journal club	III. Study design; VII. Clinical research interactions (RCR); IX. Scientific communication; XI. Translational teamwork; X. Cultural diversity; XIII. Cross Disciplinary Training
	Office hours; weekly social events (formal and informal peer mentor chat groups; ice breaker activities; movie nights; group game activities)	
Weeks 5–8	Continue mentored research project	IV. Research implementation; XI. Translational teamwork; XII. Leadership
	Community partner field experience	X. Cultural diversity; XIV. Community engagement
	Weekly journal club	III. Study design; VII. Clinical research interactions (RCR); IX. Scientific communication; XI. Translational teamwork; X. Cultural diversity; XIII. Cross Disciplinary Training
	Preparation for end of program symposium and poster presentations (2017–2019) Introduction to the Three-Minute Thesis (2020–2021)	IX. Scientific communication
	Optional learning opportunities: Issues in health care access; racism and health care; patient health experience; service projects (2019–2021)	XI. Translational teamwork; X. Cultural diversity; XIII. Cross Disciplinary Training
Weeks 9–12	Complete mentored research projects	IV. Research implementation; XI. Translational teamwork; XII. Leadership
	Weekly journal club	III. Study design; VII. Clinical research interactions (RCR); IX. Scientific communication; XI. Translational teamwork; X. Cultural diversity; XIII. Cross Disciplinary Training
	Capstone research project presentations	IX. Scientific communication
	Program feedback and reflection	XII. Leadership

### Transition to a Remote Format

Prior to 2020, students participated in all research and curriculum activities in-person at the University of Michigan Ann Arbor campus. However, due to restrictions related to the COVID-19 pandemic, the program was reformatted in 2020 to be fully remote, including participation in remote research activities. Students in the 2021 cohort also participated remotely.

Since the inception of the MICHR summer research program, learning activities have been mapped to CTSA-defined competencies. To adapt this program to a remote format, all curriculum activities were reviewed by program managers and the faculty director to determine if 1) program competencies continued to align with the new remote program structure, 2) programmatic activities could be structured in a manner that fit a synchronous, remote learning format to accommodate multiple time zones, and 3) adequate resources were available to provide equitable access for all participants and to maintain the experiential and group learning structure that was deemed critical to the success of the summer program, including participation in remote research activities. Activities that met these criteria were retained and, if needed, redesigned for remote learning. Items that did not contribute to mastery of defined translational or health disparities research skills or those that were not able to be presented in a synchronous, virtual format were replaced with learning activities that better met the program goals.

In all, 12 of the 15 activities (80%) from the in-person curriculum were retained in the remote format. Three activities were not included because the sponsoring departments ceased offering them in 2020. During program planning, we recognized that students’ access to online research tools and learning platforms would vary. To ensure that all students had fair and equitable access to the program, we contacted them before the program started and asked about their level of internet access, their access to a computer with a camera and microphone, what time zone they were in, and their familiarity with learning management systems. Adjustments were made to the curriculum activities to promote access for all students. For example, when we discovered connectivity issues, such as temporary loss of internet access for some students during Hurricane Isaias in 2020, we modified assignments to accommodate students’ needs.

Synchronous, online learning in diversity, equity, and inclusion from across the University of Michigan was made available to students as optional activities in the curriculum. In addition, regular remote office hours with the program managers, unstructured chat sessions with a peer mentor, and a variety of virtual social activities (ice breaker activities, movie nights, group game activities) were added to foster connections and strengthen peer relationships. To reduce potential online participation fatigue and enhance contact with students, activities that were previously presented in 2–3 hour in-person seminars were adjusted to be presented in multiple 1-hour segments.

Principles of instructional design were used to ensure that core learning objectives and the program’s critical hands-on learning structure were maintained [20]. Pre-recorded video lectures were not used. Instead, an emphasis was placed on developing synchronous participatory activities including group projects, student-led journal clubs, discussion groups, and role play activities. Program activities used features of online learning to enhance engagement. For example, screen sharing for participants, polls and surveys, and closed captioning were enabled for all activities. Eight of 11 activities (72%) used annotation, virtual whiteboards, or shared documents for collaborative projects; 5 of 11 activities (45%) used breakout rooms to promote teamwork. Presentations for journal clubs and the program’s capstone presentation were conducted in a live, synchronous manner with participants having the ability to raise hands to ask questions or add comments and questions to a monitored chat. Students were encouraged to have their cameras on during learning activities, but it was not a requirement.

At the beginning of this transition, few faculty members at our institution had experience with remote learning. To familiarize our faculty presenters and research mentors with the virtual platform, we held multiple, brief training sessions that provided tips on best practices for remote teaching and learning. We also provided personal technical assistance during the synchronous remote seminars.

### Data Collection

Since the program’s inception, we have developed and implemented a robust evaluation plan. Because of this, we could evaluate the impact of the remote format in comparison with the in-person format. We chose to include data from three in-person cohorts (2017–2019) and two remote cohorts (2020 and 2021). To assess the success of the new remote program structure, students were asked to complete surveys once a month during the 3-month program (three surveys total) to ensure their needs were identified and responded to in an appropriate and timely manner. This was a necessary departure from the single end-program survey structure that past in-person cohorts of students completed. Students were asked to answer eight questions about their experiences in the program. Six questions matched those from past programmatic surveys, including questions related to the relevance of the didactic sessions to students’ research activities, whether and how the didactic sessions improved understanding of the topic presented, how often students met with their summer research teams, and students’ experiences working with their faculty mentors. Two new questions were included to assess the frequency and mode of communication between the students and their mentors as well as their experience with the technology used for the remote program sessions. We also administered a survey to faculty mentors at the end of the program in the 2020 and 2021 cohorts.

We recognize that student evaluation surveys do not always provide a full picture to inform program improvement; therefore, we held focus group sessions with students in the 2020 and 2021 cohorts to supplement the survey data [21]. This structured focus group replaced previous years’ more informal face to face feedback sessions where students were asked to reflect on the strengths and weaknesses of various aspects of the program. The focus groups took place remotely during the last week of the program. Three themes were used to guide the discussions: 1) How did participation in the MICHR summer research program impact students’ short- and long-term plans for a research career, 2) how did students and mentors work together to advance research, and 3) what aspects of the program had the greatest positive impact on students’ understanding of translational and health disparities research. Students were asked 10 questions related to the overarching themes. With students’ permission, two note takers recorded the discussion and comments, and the transcripts were coded for themes by two trained coders.

### Data Analysis

To assess changes in student experience between the in-person and remote formats, scores from feedback surveys of three prior cohort years were compared to survey scores from the remote program cohorts. This analysis was conducted with a series of 5 × 1 one-way ANOVAs, with simple planned contrasts using SPSS 26 software [22]. This project was reviewed and deemed exempt by the University of Michigan Institutional Review Board.

## Results

Students from all five cohort years (*n* = 102) participated in translational and health disparities research projects during their time in the program. Seven students in the 2020 cohort and four in the 2021 cohort participated in research projects directly related to COVID-19. As seen in Table 2, the program included diverse cohorts of students from a variety of disciplines, most commonly from health-related fields such as public health, medicine, pharmacy, nursing, dentistry, and biology but also informatics, kinesiology, and biomedical engineering. Notably, significantly more students who meet the NIH criteria for disadvantaged status and Black/African American students participated in the program when it was offered remotely.

**Table 2.** Participant demographics

	2017 (*n* = 18)	2018 (*n* = 19)	2019 (*n* = 20)	2020 (*n* = 20) (remote)	2021 (*n* = 25) (remote)	χ2
*Gender*						
Female	15 (83%)	13 (68%)	15 (75%)	15 (75%)	18 (72%)	
Male	3 (17%)	6 (32%)	5 (25%)	5 (25%)	7 (28%)	
*Race*						
American Indian or Alaska Native	2 (11%)	0 (0%)	0 (0%)	0 (0%)	0 (0%)	White vs. Black, 4.45, *p* < 0.05
Asian	4 (22%)	3 (16%)	3 (15%)	3 (15%)	3 (12%)	
Black or African American	4 (22%)	4 (21%)	5 (25%)	6 (30%)	13 (52%)	
More than one race	1 (6%)	1 (5%)	4 (20%)	2 (10%)	3 (12%)	
Native Hawaiian or other Pacific Islander	0 (0%)	1 (5%)	0 (0%)	1 (5%)	0 (0%)	
Other or not reported	1 (6%)	2 (11%)	3 (15%)	4 (20%)	1 (4%)	
White	6 (33%)	8 (42%)	5 (25%)	4 (20%)	5 (20%)	
*Ethnicity*						
Hispanic or Latinx	1 (6%)	2 (11%)	3 (15%)	3 (15%)	5 (20%)	
*Disadvantaged* **						
Yes	7 (39%)	4 (20%)	6 (30%)	9 (45%)	13 (52%)	Disadvantaged vs. non-disadvantaged, 4.00, *p* = 0.05
No	11 (61%)	14 (70%)	14 (70%)	10 (50%)	12 (48%)	
Do not wish to provide	0	0	0	1 (5%)	0	

** For this program, an individual was considered disadvantaged if they met two or more criteria of the NIH definition including, but not limited to homelessness, being in the foster care system, eligible for Federal Free and Reduced Lunch program, and eligible for or receiving a Pell grant [23].

### Survey Results

Mean scores for each item and cohort year for the feedback survey are shown in Table 3 (rating scale of 1 – strongly disagree to 5 – strongly agree for each item). Overall, students in both the in-person and remote program cohorts rated their experience very high, with no question receiving a score less than 4.0. However, there were notable differences for students between the remote and in-person formats. Planned comparisons showed that students in the remote cohorts gave significantly higher ratings for three items: 1) the program encouraged student interest in pursuing a clinical, translational, or health disparities research career, 2) students learned information that would be useful in their future career, and 3) students would recommend the program to others. Additionally, most faculty mentors in the remote cohorts indicated that they were satisfied or very satisfied with the virtual platforms as a form of communication, though many preferred an in-person program over a virtual program.

**Table 3.** Mean feedback survey scores for each cohort year

Item	2017	2018	2019	2020 (remote)	2021 (remote)	*p*
I learned information in the summer program that will be useful to me in my future career	4.78	4.38	4.57	4.78	4.87	*t*(40.80) = 2.40, *p* < 0.05
The summer program encouraged my interest in pursuing additional training in clinical, translational, or health disparities research	4.44	4.38	4.29	4.89	4.87	*t*(27.44) = 4.18, *p* < 0.01
Having notes, slides, and supplemental readings available in Canvas was helpful for me	4.28	4.25	4.50	4.50	4.43	*t*(76) = 0.76, *p* = ns
I would recommend the summer program to my colleagues	4.56	4.50	4.64	5.00	4.83	*t*(22.29) = 2.82, *p* < 0.01
The mentored research experience helped me gain new knowledge about the clinical, translational, and health disparities research process	4.67	4.50	4.69	4.89	4.64	*t*(47.67) = 1.22, *p* = ns
My research mentor encouraged my participation and critical thinking	4.72	5.00	4.69	4.78	4.73	*t*(62.59) = −0.58, *p* = ns
I was able to dedicate adequate time to my mentored research project	4.72	4.88	4.69	4.78	4.54	*t*(68.43) = −0.88, *p* = ns
I was able to spend adequate time with my research team or mentor	4.56	4.88	4.54	4.50	4.77	*t*(74) = −0.12, *p* = ns

### Focus Group Results

Focus group sessions were held at the end of the program with students in the 2020 (*n* = 18) and the 2021 cohorts (*n* = 19). Five overarching themes emerged: 1) interest in the program, 2) impact of the program on students’ plans to continue with a career in research, 3) the effects of mentorship on students’ research experiences, 4) attitudes toward the program, and 5) the remote aspects of the program.

Students cited several reasons for their interest in the program including gaining research experience before applying to graduate programs, interest in learning more about health disparities and translational research, and the opportunity to explore translational research as a future career (“I wanted to have research experience before starting my PhD. [That’s] hard to find for master’s students.”). When asked how participating in the program affected their plans for continuing in research, students said it had a positive impact on their long- and short-term career goals as well as their view of research as a career path (“I didn't think I would do research in my second year of med school…I plan to continue working with my mentor…which wasn’t my original plan.”). For some students, experience in the summer research program confirmed their choice of career, while others said the program exposed them to new career possibilities and was a catalyst to changing their career paths (“I realized I wanted to focus on clinical research as a career, not just to supplement my applications to medical school and residency.”). When discussing their mentor/mentee relationships, students said their mentors exposed them to new career possibilities, helped them establish professional social networks and career goals, and provided support and guidance (“[It was] interesting to talk with my mentor and [get her] thoughts about choosing a specialty. It was nice to see a different perspective.” “[My mentor] helped me find resources…and connected me with people who can help me with my career.”). In fact, several students said they planned to continue working with their mentor after the program ended, which was not their original intent. Lastly, students described the opportunity to develop research communication skills, the variety of learning experiences, and developing relationships with mentors as positive aspects of the program. Students also suggested potential program improvements and made recommendations for students considering applying to the program in the future.

We asked students to share what they liked and did not like about the remote format. Several said they missed having social interactions with their peers and expressed a desire to be more connected (“It would be nice to have a few more sessions like the social hours where students in the program could talk to each other. I’m sure it was much different when the program was in person but having that kind of touchpoint and being able to interact with each other more rather than being in a presentation together.”). Students also expressed a need for more bonding time (“I really enjoyed the opportunities that we did have to see what everyone else was working on and get to hear about everyone else’s research. I wish we would have had a little bit more time for collaboration like that… I think [being online] put some constraints on that”). This feedback suggests that while students liked the program, the online format constrained their ability to form a sense of community with students in their cohort and with their research mentors.

## Discussion

Like other education programs in the era of COVID, moving our summer program online came with challenges. However, it also brought us the opportunity to recruit and train a more diverse group of students. The remote format allowed students greater access to training and resources they might not otherwise have had by eliminating barriers to participation, such as the cost of relocating to our university for the duration of the program, and providing increased access to a rich network of faculty mentors [24]. In addition, we were able to reevaluate our curriculum to ensure it provided up-to-date training in clinical and translational science [18] and remained current with trends in remote learning in medical education [25].

Because the program already included a robust evaluation plan, the evaluations we have conducted since 2017 enabled us to directly compare student perceptions of the remote format to the in-person format. Overall, students in both the in-person and remote cohorts viewed the program favorably, with students in the remote cohorts indicating their program experience increased their interest in pursuing a career in health disparities research, helped them learn new information that would be useful in their future career, and that they would recommend the program to others *significantly more than* those in the in-person cohorts. We also saw a significant increase in the diversity of the students in our program, both in terms of race and disadvantaged status. The ability for students to participate remotely helped them see our program as a viable and attainable training opportunity, and we anticipate that the positive impact of the program will feed into the diversity of the CTR workforce.

### Lessons Learned

Several important lessons were learned from the process of moving a highly successful in-person program to an equally successful remote program.

1. Mapping curriculum learning activities to established competencies was an important tool in the design of our program, whether the learning was being conducted in-person or remotely. This strategy allowed us to design activities that met defined learning objectives and program goals [20,26]. Not all program learning components needed to be moved to an alternate learning format to maintain programmatic goals. When determining which activities to move to alternate formats, we selected those that matched defined program competencies **and** could be measured through a structured program evaluation plan.
2. In our program, web access, time zone changes, and other technical issues posed barriers to participation for some students. Research has shown that these types of issues may pose disproportionate barriers for underrepresented students [24,27]. When developing program activities, we sought out ways to promote equity and minimize barriers. This included encouraging, but not requiring, camera use during synchronous remote activities, including both written and verbal learning activity instructions, and providing program content in multiple ways (including asynchronously) to maximize access and inclusion [20,27].
3. In reviewing our curriculum, we realized that learning activities that worked well in two- or three-hour classroom seminars were too long to be presented in a remote format. To promote inclusion and engagement for all program participants, we divided curriculum activities into smaller segments. Taking frequent breaks to answer questions or provide clarity for activities also encouraged inclusion and engagement [21].
4. When creating our curriculum, whether in-person or remote, we incorporated varied teaching strategies and learning technologies to encourage an inclusive learning environment and meet students’ needs [20,28]. We included multiple strategies including synchronous lectures with closed captioning, learning supplements in written and audio formats, synchronous online platforms for instructor-led and group learning activities, Google forms for collaborative writing, and institutional learning management systems to post program assignments, provide instructor feedback, and host electronic portfolios to promote accessibility and inclusion [28].

## Conclusion

The process of redesigning the MICHR summer research program was unanticipated, but it allowed us to meet students where they are and examine how equity and inclusion factor into and impact our program. In future years, we are thinking more critically about what would be most equitable for all students who may be interested in participating in our program, but who may have experiences or circumstances that may deter or prevent them from participating in an in-person or residential program at the University of Michigan. Students’ comments about longing to form community among their cohort or meet with their research mentor and team in-person echoed our own impressions that a remote connection cannot replace an in-person experience in all cases. In the summer of 2022 and beyond, we will explore potential hybrid approaches, with some students participating in in-person research and others participating in remote research, and all students participating in remote learning sessions and activities together. In the future, we will examine the long-term impact that the virtual program had on students’ publications and career trajectory.
